# User Access Management Based on Network Pricing for Social Network Applications

**DOI:** 10.3390/s18020664

**Published:** 2018-02-24

**Authors:** Fuhong Lin, Zhibo Pang, Xingmin Ma, Qing Gu

**Affiliations:** 1 School of Computer and Communication Engineering, University of Science and Technology Beijing (USTB), Beijing 100083, China; maxingmin1983@163.com; 2 ABB Corporate Research, Forskargränd 7, SE-721 78 Västerås, Sweden; Pang.zhibo@se.abb.com; 3 School of Mechanical Engineering, University of Science and Technology Beijing(USTB), Beijing 100083, China; qinggu@ustb.edu.cn

**Keywords:** resource management, network pricing, unascertained membership degree

## Abstract

Social applications play a very important role in people’s lives, as users communicate with each other through social networks on a daily basis. This presents a challenge: How does one receive high-quality service from social networks at a low cost? Users can access different kinds of wireless networks from various locations. This paper proposes a user access management strategy based on network pricing such that networks can increase its income and improve service quality. Firstly, network price is treated as an optimizing access parameter, and an unascertained membership algorithm is used to make pricing decisions. Secondly, network price is adjusted dynamically in real time according to network load. Finally, selecting a network is managed and controlled in terms of the market economy. Simulation results show that the proposed scheme can effectively balance network load, reduce network congestion, improve the user's quality of service (QoS) requirements, and increase the network’s income.

## 1. Introduction

Many aspects of social networks have been studied [1,2,3]. To guarantee quality of service (QoS) for users, a considerable amount of resources is required. Many kinds of wireless networks have been proposed and deployed in recent years, and many can provide transmitting services. In the end, users must choose a network to connect to, but how a user chooses a network needs to be optimized. In terms of network resource management, it is difficult to increase profits as well as guarantee effective load balance and reduce network congestion. One solution is proposed by Alsharif et al. [4] and Luo et al. [5]. Essentially, they integrated all kinds of wireless networks into one network that users can access. However, a network selection process still exists. Usually, various resource management schemes are used to solve this problem. Mostaani et al. [6] presented a pricing scheme that can optimize resource management in cognitive radio networks. However, they focused on single network pricing and did not consider a multi-network environment. Based on reinforcement learning, Chenwei et al. [7] proposed a heterogeneous wireless network resource management algorithm. The shortcoming of this was that the congestion rate was too high. Chanak et al. [8] presented a fuzzy classification management scheme in a large-scale wireless sensor network. This scheme lowered the congestion rate. However, the load threshold was too low to be used in the actual environment. Tsiropoulou et al. [9] proposed an SC-FDMA uplink resource allocation scheme for the Papavassiliou investigation and classification in a wireless network. In this scheme, the congestion rate and network load threshold were still unable to meet the actual environment requirements. Amin et al. [10] presented a controlling algorithm that could evaluate global resources with heterogeneous wireless networks. However, this scheme also had a high congestion rate. Hang et al. [11] proposed an interference management algorithm in a mixed, small, self-organizing cellular network, but the congestion rate could still be improved. Zhang et al. [12] presented a congestion mitigation scheme but did not consider a heterogeneous network environment.

In sum, the algorithms mentioned above have made significant achievements in improving wireless network performance by optimizing the resource management scheme. However, network performance in terms of congestion rates or load balance can still be further optimized. In this paper, based on network pricing and the rate of unascertained membership, we propose a wireless network access management scheme to lower the congestion rate, achieve load balance, and increase the network revenue. In the proposed scheme, different kinds of wireless networks work cooperatively. Using an unascertained membership degree algorithm, the network price is adjusted according to the real-time changes in the following parameters: network bandwidth, load, and the number of users. Based on the network price, users choose an appropriate network. Numerical simulation shows that the algorithm works to decrease network congestion, achieve load balance, and improve network revenue when there is high load and a large number of users.

The sections are organized as follows. In Section 2, the system environment is briefly described. In Section 3, the access scheme is proposed and analyzed. Numerical simulation is carried out in Section 4, and conclusions are drawn in Section 5.

## 2. System Environment Description

**Figure 1:**
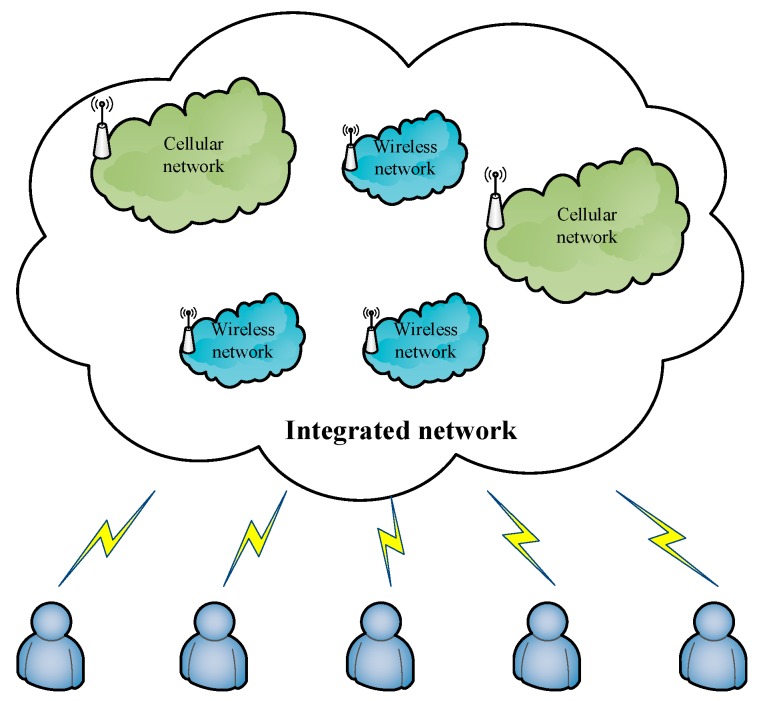
The integrated network model.

The following scenario is considered in this paper. As shown in Figure 1, the integrated network is composed of three wireless networks and two cellular networks. The wireless network in Figure 1 could be a WLAN or a wireless network that is organized by a micro-base station. A user can receive service by accessing either. The difference between the wireless network and cellular network is that the bandwidth is smaller and the coverage area is larger in the cellular network. The radiation ranges of the cellular network and wireless network are 2 km and 0.1 km, respectively. The initial basic bandwidth provided by the wireless network is about ten times that of the cellular network.

In the integrated heterogeneous network, since user access is affected by multiple factors, user access strength is different. There are four main factors: network signal coverage intensity, network load, network available bandwidth, and regional network pricing. 

In general, the network signal coverage intensity is one of the determinants of network availability. The higher the signal strength, the stronger the availability, but the higher the network pricing is. Usually, users tend to access the network whose price is the lowest. If increased numbers of users access the cheaper network, then network congestion and load imbalance problems are easily produced. In the proposed scheme, the four factors mentioned are set as the inputs of the unascertained algorithm. Using this algorithm, the network decision is obtained, further providing effective guidance for the user in choosing the optimal network from which to receive service.

## 3. Algorithm Description

Unascertained is a new kind of uncertainty that is different from randomness and fuzziness, and this uncertainty is very common. The processing of unascertained information cannot be processed according to exact information; the information must be treated as uncertain. Because it is different from random information and fuzzy information, it cannot be handled according to methods of random information and fuzzy information. The unascertained degree of membership is a new method to solve the ascription of unascertained information.

In 2004, Liu Kaidi, Cao Qingkui, and Pang Yanjun put forward the unascertained set for the first time and applied it to ship engine fault diagnosis [13]. In [13], a new set of uncertainty is defined, called the unascertained set. Its characteristic is the measurement criteria explicitly in the definition of membership function. The unascertained set has a property that is usually set, and the unascertained logic system can deal with unascertained information. Due to the introduction of the concept of weight index classification, the algorithm can be reliably interpreted. Therefore, the method of unascertained membership is also applicable to the comparison and judgment of the parameters of different network access standards in heterogeneous networks.

Let $U=\left\{x_{1},x_{2},\cdots ,x_{n}\right\}$, where *x_i_* represents the *i*-th object. The property or state provided by the object is designated as *F* and called the property or state space $F_{i}\;\left(i=1,2,\ldots \right)$ is the *i*-th specific property or state in *F*, recorded as $F_{i}\in F$ [14]. All subsets of *F* compose the set *E*. If $A_{i}\in E$, then $A^{-1}\in E$. In other words, *E* is closed to the complement operation. If $A_{i}\in E\left(i=1,2,\ldots ,k\right)$, then $\overset{k}{\underset{i=1}{\cup }}A_{i}\in E$, $A^{-1}\in E$ In other words, *E* is closed to a finite union operation, and *E* is called an algebra set, which is based on *F*. If *E* is an algebra set, *E* is close to the complement operation and the finite union operation. If *E* is closed for countable collections $A^{-1}\in E$, then $\overset{\infty }{\underset{i=1}{\cup }}A_{i}\in E$. *E* is an σ algebra set. Obviously, if *E* is an σ algebra, then any complementing set of *E* is in *E*, and the union of countable sets of *E* is also in *E* [15].

### 3.1. The Measure of Uncertainty and the Uncertainty Set

*U* is set as the domain and *F* as the property of space on *U*. $\left\{F_{1},F_{2},\ldots ,F_{k}\right\}$ is a division of *F*. The property or state of object *x* in *U* is usually affected by many factors [16]. The impact of these factors is the attribute or index. There are m kinds of specific attributes $I_{1},I_{2},\ldots ,I_{m}$ affecting the property or state of the object *x*, where $I=\{I_{1},I_{2},\ldots ,I_{m}\}$ is called the attribute space on the domain *U*. Arbitrarily given $x_{i}\in U$, the observation value of the *j*-th attribute can be measured in detail and called $x_{ij}$. The problem is how to define the property of $F_{k}$, considering the object *x_i_* with the observed value $x_{ij}$, and how to describe it quantitatively [17]. However, it is difficult, or even impossible, to accurately quantify the property with incomplete information or uncertain conditions.

However, using the problem background and prior knowledge, the decision maker can quantify the property. In fact, a different property is introduced by a different quantity. From this point, it can be measured. To carry out measurement, we need to establish a measurable space and then take the measurement according to relevant criteria. We have discussed in Section 2 that *E* of *F* can be generated by a division of a given property space *F*. Therefore, it can be constructed in the form of a measurable space in order to quantitatively describe the property of the observed object with a specific property of *E*. Therefore, whether or not a reasonable measurement can be carried out is decided by the possibility of constructing a measurement criterion in the space (F, E) [18]. The following measurement criterion is proposed [19].

**Definition 1.**  *Given the arbitrarily fixed objects* $x_{i}\in U$ *and arbitrarily fixed attributes* $I_{j}$*,* $x_{ij}$ *is the observed value of* $x_{i}$ *according to* $I_{j}$*, If there is a mapping* μ *with any* A∈E *satisfying the constraints presented in Equations (1)–(3), then* $\mu_{A}\left(x_{ij}\right)$ *is the measurement function of the uncertainty in measurable space* (F, E)*.*

(1) $$0\leq \mu_{A}\left(x_{ij}\right)\leq 1$$

(2) $$\mu_{\overset{}{\underset{l=1}{\cup }}A_{l}}\left(x_{ij}\right)=\sum_{l=1}^{}\mu_{A_{l}}\left(x_{ij}\right)$$

(3) $$\mu_{F}\left(x_{ij}\right)=1$$

*where $i=1,2,\ldots ,n,\;j=1,2,\ldots ,m,\;A_{l}\in E,\;l=1,2,\ldots$.*

Equation (1) is a nonnegative bounded constraint. Equation (2) is an additivity constraint; Equation (3) is a normalization constraint. These three constraints are the criteria that must be obeyed in the usual measurement [20]. Decision makers are often limited to the measurement function under the condition that the information is not sufficiently complete. As such, μ is unascertained. In addition to the necessary prior knowledge, it also contains the decision maker’s preferences, requirements, and other subjective factors [21].

**Definition 2.**  *If U is a domain, F is the property of space on U,* (F,E) *is the measurable space on U,* $\mu_{A}\left(x\right)$ (x∈U,A∈E) *is the uncertainty measurement function on the measurable space* (F,E)*, and* $\left(F,E,\mu_{A}\left(x\right)\right)$ *is called the uncertainty measure space on domain U*.

**Definition 3.**  *If U is a domain and* $\left(F,E,\mu_{A}\left(x\right)\right)$ *is the uncertainty measure space on U, then* $\mu_{A}\left(x\right)$ *is the membership function to determine an uncertain subset* $\tilde{A}$ *of algebraic E in domain U, and* $S\left(\tilde{A}\right)$ *is the supporting set of* $\tilde{A}$*, which is shown in Equations (4) and (5). If* $x\in S\left(\tilde{A}\right)$*, x is the support point of* $\tilde{A}$ *and A is preimage set of* $\tilde{A}$ *on E.*

(4) $$\tilde{A}=\left\{x|0\leq \mu_{A}\left(x\right)\leq 1\;,\;\forall x\in U\;,\;A\in E\right\}$$

(5) $$S\left(\tilde{A}\right)=\left\{x|0<\mu_{A}\left(x\right)\leq 1\;,\;\forall x\in U,\;A\in E\right\}$$

Any given A∈E, $\tilde{A}$ can be treated as a fuzzy subset of the domain *U*. For any fixed x∈U, $\mu_{A}\left(x\right)$ is a function on σ algebra *E* [22]. As such, $\mu_{A}\left(x\right)$ is defined as two element functions on U×E. For any x∈U, $0\leq \mu_{A}(x)\leq 1$, a fixed *x*, and $A_{i}\in E$, i=1,2,…,k, $\mu_{A_{i}}\left(x\right)$ satisfies the constraint presented in Equation (6).

(6) $$\{\begin{array}{l} \mu_{\overset{k}{\underset{l}{\cup }}A_{l}}\left(x\right)=\sum_{l=1}^{k}\mu_{A_{l}}\left(x\right)\\ A_{i}\cap A_{j}=\varphi ,\left(i\neq j\right)\\ \mu_{F}\left(x\right)=1 \end{array}$$

Therefore, in σ algebra, $\mu_{A}\left(x\right)$ meets the criteria for the additive criterion because space F∈E, $\mu_{A}\left(x\right)$ meets the normalization criterion.

### 3.2. The Uncertainty Measurement Function

Although the uncertainty measurement function is defined on the measurable space (F,E), and the measurement criterion is defined according to the definition of the measurement function, the definition is non-constructive. In practical applications, decision-making is specifically constructed by background, relevant domain knowledge, prior knowledge, and personal needs and preferences. In order to facilitate the application, there are several construction methods commonly used for measurement function.

**Figure 2:**
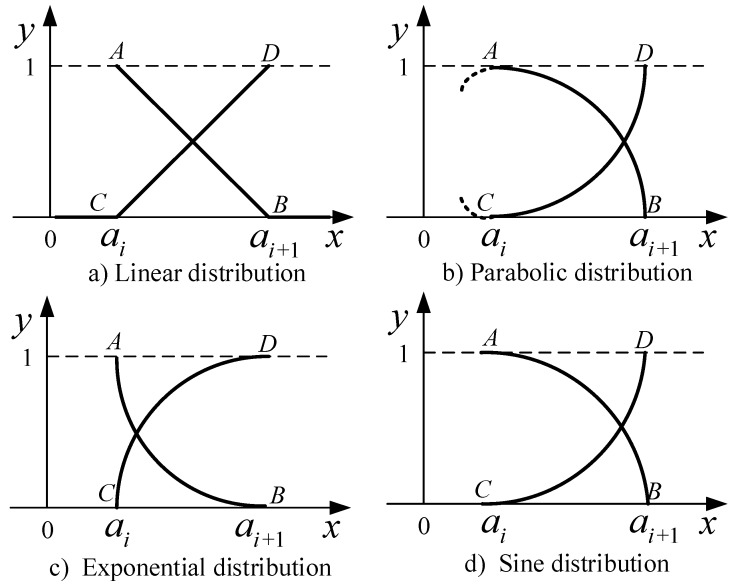
Grades of unascertained membership.

If a partition of the property space *F* contains *k* specific properties, we can insert k−1 points $a_{1},a_{2},\ldots ,a_{k-1}$ (these can be equally inserted or unequally inserted) on the distributed interval. Where *k* is the number of the partition of the *F* in the property space [23], assuming that the value of the property at the left of the point $a_{i}$ is in the *i*-th state, when the attribute value is increasing from $a_{i}$ to $a_{i+1}$, the *i*-th state of the attribute is gradually weakened. The degree of the i+1 state of the attribute observation is reduced to zero. At the same time, when the observed value increases from $a_{i}$ to $a_{i+1}$, the degree of the i+1 state of the attribute observation increases from zero to *1*. We should pay attention to the observed state changes in the relevant reference point in the vicinity of the sensitive area on the property values. Based on the severity of the state change, as mentioned in [15], decision makers can use a straight line, two curves, a sine curve, an exponential curve, and other curves connecting to construct the concrete expression of the measurement function, which is shown in Figure 2.

The corresponding expressions of the uncertainty measurement function are in Equations (7)–(10):

(7) $$\left\{ \begin{array}{l} \mu_{i}(x)=\left\{ \begin{array}{l} \frac{-x}{a_{i+1}-a_{i}}+\frac{a_{i+1}}{a_{i+1}-a_{i}}\;a_{i}<x\leq a_{i+1}\\ \;0\;x>a_{i+1} \end{array}\right.\\ \mu_{i+1}(x)=\left\{ \begin{array}{l} \;0\;x\leq a_{i}\\ \frac{x}{a_{i+1}-a_{i}}-\frac{a_{i}}{a_{i+1}-a_{i}}\;a_{i}<x\leq a_{i+1} \end{array}\right. \end{array}\right.$$

(8) $$\left\{ \begin{array}{l} \mu_{i}(x)=\left\{ \begin{array}{l} 1-{\left(\frac{x-a_{i}}{a_{i+1}-a_{i}}\right)}^{2}\;a_{i}<x\leq a_{i+1}\\ \;0\;x>a_{i+1} \end{array}\right.\\ \mu_{i+1}(x)=\left\{ \begin{array}{l} \;0\;x\leq a_{i}\\ {\left(\frac{x-a_{i}}{a_{i+1}-a_{i}}\right)}^{2}\;a_{i}<x\leq a_{i+1} \end{array}\right. \end{array}\right.$$

(9) $$\left\{ \begin{array}{l} \mu_{i}(x)=\left\{ \begin{array}{l} 1-\frac{1-e^{x-a_{i}}}{1-e^{a_{i+1}-a_{i}}}\;a_{i}<x\leq a_{i+1}\\ \;0\;x>a_{i+1} \end{array}\right.\\ \mu_{i+1}(x)=\left\{ \begin{array}{l} \;0\;x<a_{i}\\ \frac{1-e^{x-a_{i}}}{1-e^{a_{i+1}-a_{i}}}\;a_{i}<x\leq a_{i+1} \end{array}\right. \end{array}\right.$$

(10) $$\left\{ \begin{array}{l} \mu_{i}(x)=\left\{ \begin{array}{l} \frac{1}{2}-\frac{1}{2}\sin\frac{\pi }{a_{i+1}-a_{i}}\left(x-\frac{a_{i+1}-a}{2}\right)\;a_{i}<x\leq a_{i+1}\\ \;0\;x>a_{i+1} \end{array}\right.\\ \mu_{i+1}(x)=\left\{ \begin{array}{l} \;0\;x\leq a_{i}\\ \frac{1}{2}+\frac{1}{2}\sin\frac{\pi }{a_{i+1}-a_{i}}\left(x-\frac{a_{i+1}-a_{i}}{2}\right)\;a<x\leq a_{i+1} \end{array}\right. \end{array}\right.$$

In the above function expression, the value of $\mu_{i}(x)$ at the left point is 0. The images of $\mu_{i}(x)$ at $\left[a_{i+1},a_{i+2}\right]$ and $\mu_{i+1}(x)$ at $\left(a_{i},\;a_{i+1}\right]$ are the same. Additionally, the images of $\mu_{i+1}(x)$ at $\left[a_{i-1},a_{i}\right]$ and $\mu_{i}(x)$ at $\left[a_{i},\;a_{i+1}\right]$ are the same. The value of $\mu_{i+1}(x)$ at the left point is *0*.

The above proposed unascertained membership function $\mu_{i}(x)$ (i=1,2,…,k) is defined in (−∞,+∞). The linear unascertained measurement function is used in the paper. 

**Figure 3:**
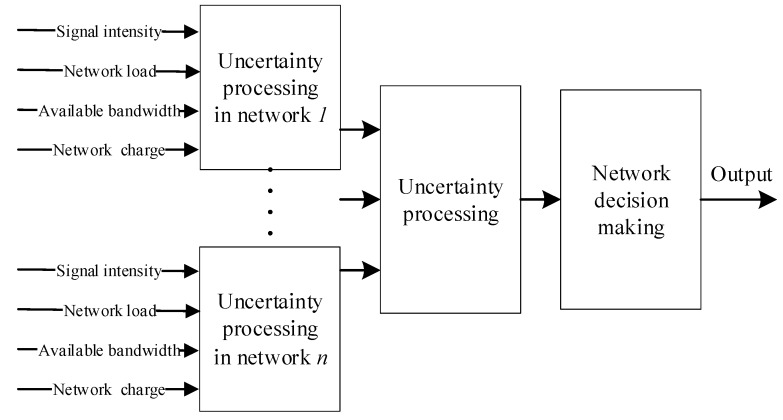
The uncertainty membership processing procedures.

For a heterogeneous network, the unascertained membership degree [15,24,25,26] method works as follows. (1) Compare and judge each of the parameters of different networks. (2) Make the common characters into an unascertained membership degree and turn the input parameters into suitable values using the unascertained membership degree function. (3) According to the unascertained membership degree rules, delete the parameter unascertained. Then, use the outcomes to make a network access judgment and control the user’s behaviors. In our proposed scheme, the unascertained membership degree input consists in the four parameters mentioned in Section 2, which are network signal coverage intensity, network load in the region, network available bandwidth in the zone, and regional network pricing. Using the proposed unascertained membership degree method, the optimal network access and the optimal user’s behaviors can be determined, as shown in Figure 3.

**Figure 4:**
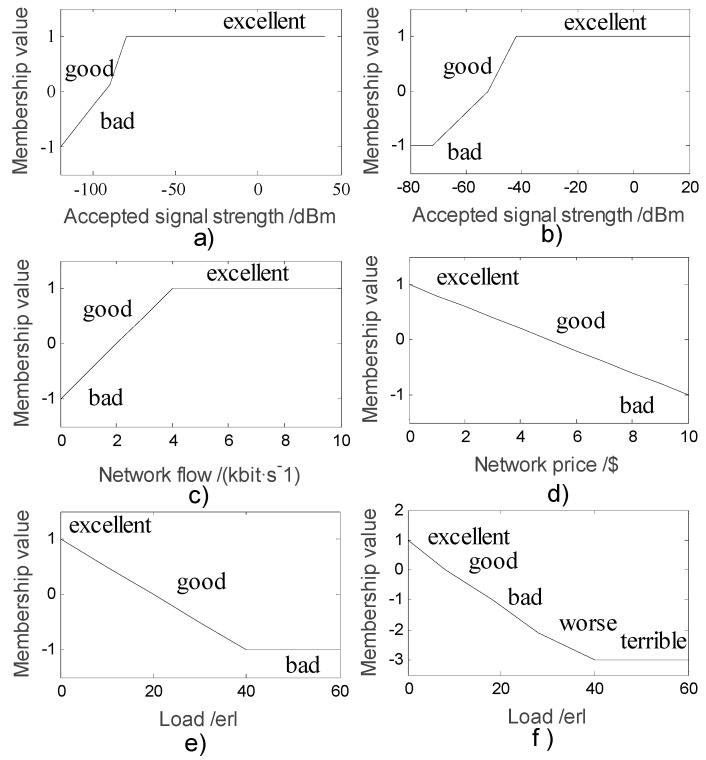
(**a**) Cellular network signals membership function; (**b**) WLAN (wireless local area network) receiving signals membership function; (**c**) network traffic membership function; (**d**) collect fees membership function; (**e**) cellular network load membership function; (**f**) WLAN load membership function.

The output of the membership function can be defined as excellent, good, bad, worse, or terrible. Moreover, the uncertainty function is determined as F∈(1,0,−1). According to the network environment proposed in Section 2, the membership functions are given in Figure 4 by considering the four parameters.

There are three WLAN access points and two cellular network access points that are indexed as *i*, i∈[1,5]. The four parameters are indexed as *k*, k∈[1,5], respectively. The network fuzzy steady-state values are determined as shown in Equation (11).

(11) $$M_{i}=\sum_{k}^{4}M_{ik}$$

where $M_{ik}$ is the *k*-th fuzzy value of the *i*-th user. $M_{i}$ represents the total fuzzy steady-state value of the *i*-th user. All the fuzzy steady-state values are calculated for each user and are then compared with each other. When the fuzzy steady-state value of a user Meng_now is lower than the preset threshold M_threshold that is shown in Equation (12), the user switches to another network for service.

(12) Meng_now<M_threshold

In order to avoid frequent switching in a short period of time, the following rule is established. When the fuzzy steady-state value of the switching target network Meng_target is greater than the value of Meng_now that is shown in Equation (13), then switching may occur.

(13) Meng_target>Ment_now

### 3.3. The Network Pricing

**Figure 5:**
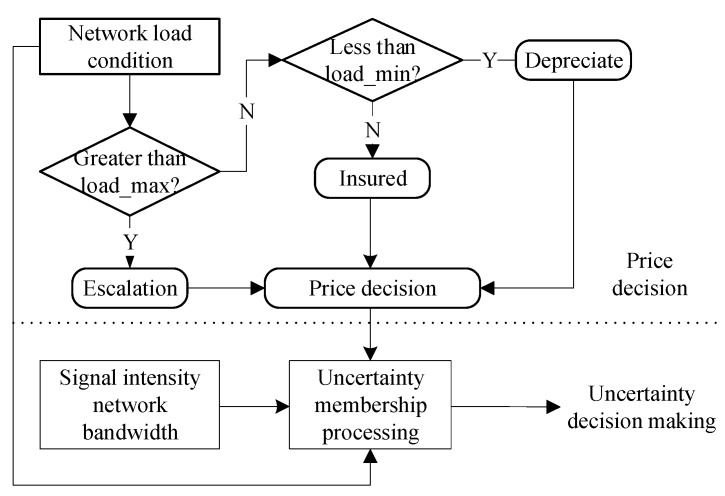
The network pricing controlling process.

The network controlling algorithm is divided into two parts, which are the pricing decision and the unascertained membership decision. The threshold values of the price decision are load_max and load_min. The network control pricing variation of a step is Δ*P*. If a network detects that there is a large number of users in it and that the real-time load_now is above the threshold load_max, then its bandwidth resource is constrained and there is a higher probability that the network becomes congested. The network increases its price with [(load_now−load_max)ΔP] to reduce the unascertained stable value. This action can prompt users to choose another network to access. By contrast, if load_now smaller than the threshold load_min, the network decreases its price with [(load_now−load_max)ΔP] to increase the unascertained stable value. If the load is between the two thresholds, the network maintains its price. After network pricing has been decided, together with the three other factors, they enter into the unascertained part of the process. The unascertained decision is thus determined, thereby determining the user’s behaviors in choosing the optimal network to access. The whole process is introduced in Figure 5.

## 4. Numerical Simulation

In this section, based on the proposed scheme, the system performance evaluation is carried out. The simulation experiment in this paper is implemented on matlab 2014 (The MathWorks, Inc., Natick, MA, USA). Computer processor: Intel (R) Core (TM) i5-3210M CPU @ 2.5 GHz; memory: (8.00 GB), 64 bit operating system. The simulation parameters are listed in Table 1. In a community, there are two kinds of services provided: a cellular network and a wireless network. The cellular network is WDMA (wideband code division multiple access), and the wireless network is an 802.11b network. Users can receive services from either of them. Users are distributed in the network coverage area, independently moving in any direction at a speed of E[v]=40 m/s, which is evenly distributed. The arrival rate of these system services (including voice and data services) can be considered as a Poisson distribution [27] with a mean of λ=6/hour, and service duration is exponentially distributed with a mean of v=20 s. The average traffic per user is about 0.2 erl. Supposing the load threshold of the integrated network is 10 erl, the load_max is 7 erl, and the load_min is 3 erl. The initial price for cellular and wireless networks are both 5 units/erl. 

With the increase in the number of users, the probability of cellular network congestion changes. The integrated network load equals the sum of each user’s communication load, and each user accords with Equation (14).

*User_communication_rate* = *λ* × *v* (14)

In Equation (14), λ is the user business arrival rate, and v represents the users’ average service time.

In [7], the authors claimed that communication services can be classified as either business data or voice service. The data service follows a normal distribution with a mean of 0.2 and a variance of 0.2, and the bandwidth of the data traffic is 2.5 times that of the voice business. The authors of [9] showed that the initial network load threshold can be set at 7 erl and changed with the network load in real time. The network load_max threshold can be set at 20 erl.

**Figure 6:**
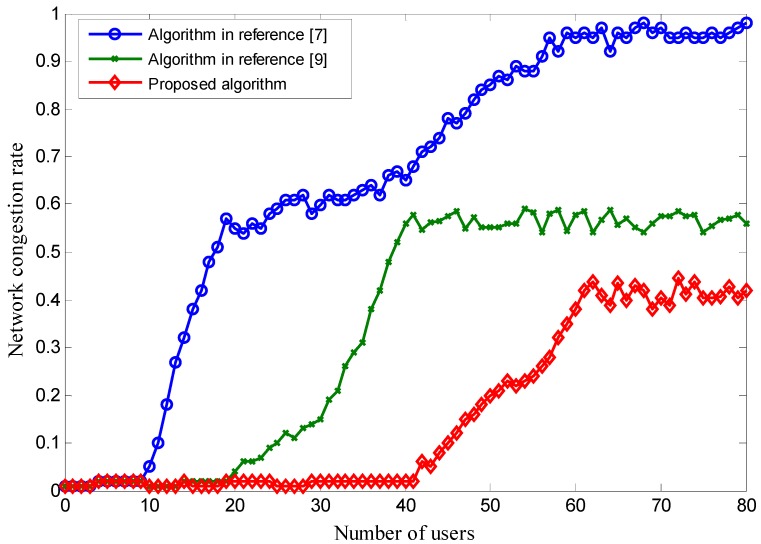
The integrated network congestion rate with smaller wireless network bandwidth.

As shown in Figure 6, when the number of users increases, the proposed scheme works better than the schemes proposed in [7,9]. The reason is that the network pricing controls the network users’ access behavior. When too many users access the network, the price is increased, the membership function’s unascertained lower steady-state value is decreased, and lastly the integrated network steady-state value is decreased. The user then chooses the network that has the higher steady-state value, which finally reduces the integrated network load and keeps the network congestion rate below 50%. Due to the network loading threshold being fixed, the scheme in [7] can easily result in a higher congestion rate when the amount of users increases. When the number of users exceeds 30, the network congestion rate is higher than 50%. Owing to the initial network load threshold being lower in [9], the congestion probability is lower than the scheme proposed in [7] but higher than in our proposed scheme.

**Figure 7:**
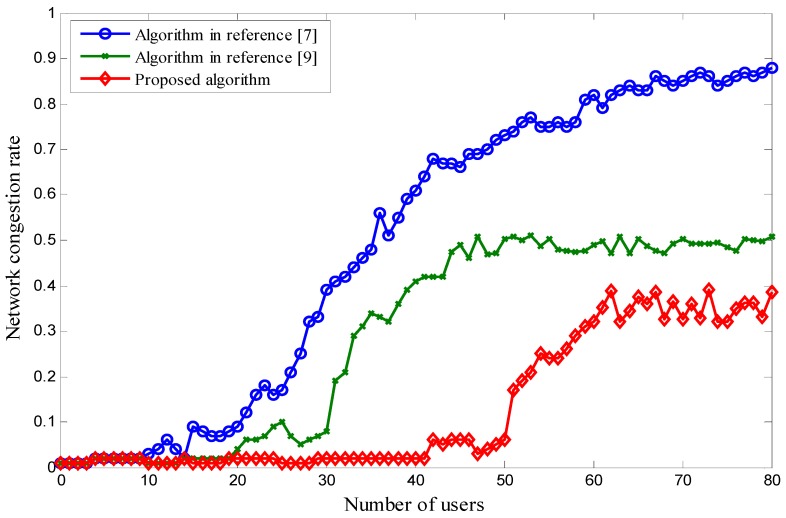
The integrated network congestion rate with larger wireless network bandwidth.

Further, we set another scenario in which the bandwidth of wireless networks are 4 Mbit/s, 4 Mbit/s, and 5 Mbit/s. The reason why we set these values is that we want to show the trends of network congestion changes. Again, we can simulate the network congestion rate using these three algorithms. The outcome is shown in Figure 7. We can see that the trends of the network congestion rate are similar to the ones in Figure 6. The difference is that, for a certain number of users, the congestion rate is lower. The reason for this is that the wireless network bandwidth is set larger. There is hardly any congestion when the number of users is smaller than 50 in our proposed scheme.

**Figure 8:**
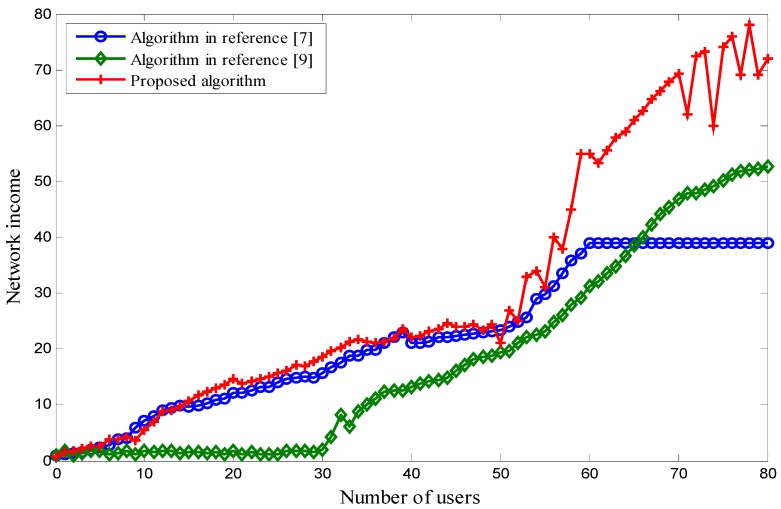
The integrated network income.

The network revenue increases with the increase in the number of users, which is shown in Figure 8. For a user *i*, assuming the communication load is *T_i_* and the network price is *p*, the network income generated by user *i* is *T_i_* × *p*. There are *n* users in total on the network at this time. Therefore, the total network income is $\sum_{i-1}^{n}$*T_i_* × *p*. Comparing the three schemes, our proposed scheme has a higher income than the other two when the number of users is more than 55. The reason is that, in our scheme, although the network load is close to the threshold and the network congestion rate is increased when the number of users is greater than 55, it increases the network price by decreasing the number of access users. Therefore, network revenue is guaranteed. In [9], the network price remains constant, and the network revenue increases with the increase in the number of users. However, there is a threshold in terms of the number of users. As such, the income becomes stabilized when the number of users reaches the threshold value. In [7], the network load threshold and the network price are fixed. Therefore, the network revenue does not change when the number of users is more than 60 at one time. The reason for this is that the network capacity is limited.

**Figure 9:**
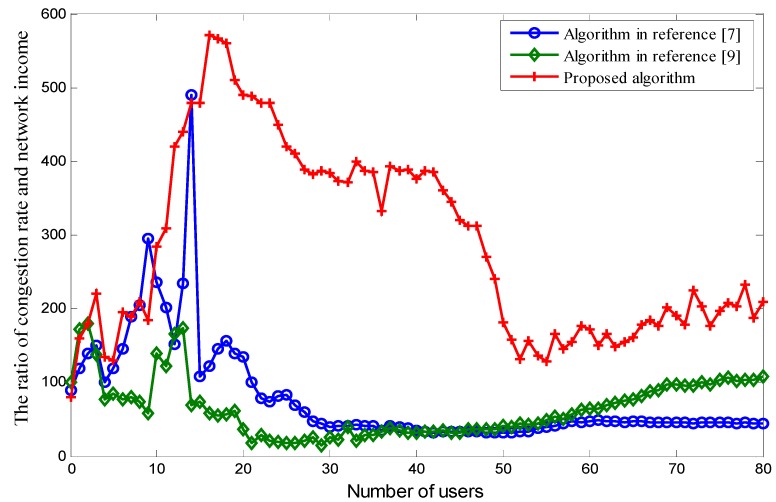
The ratio of congestion rate and network income.

Figure 9 shows the ratio of the congestion rate and network income under a different number of users for the three algorithms. In this simulation, the congestion rate is a negative factor for the network, while the network income is a positive factor. The ideal situation for a given network is where congestion rate is the lowest and network income is highest. From Figure 9, we can see that our proposed algorithm has the highest ratio for most situations. An exception is that there are two singular points at which the performance of our proposed algorithm is inferior to the performance using the algorithm proposed in [7]. Analyzing these further, we can conclude that these two singular points are abnormal and result in a flawed scenario. The two singular points are caused by random fluctuations in the system. They do not have actual physical meaning, so they can be ignored. As such, we can ignore these singular points.

**Figure 10:**
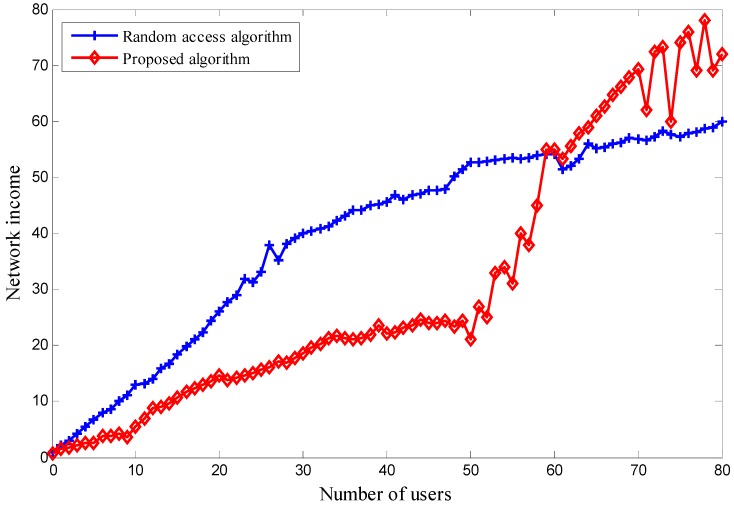
The ratio of congestion rate and network income.

We can also simulate the following scenario. No special scheme is employed and the users randomly access either network to receive service. The network income is based on the different number of users. The outcome is shown in Figure 10. The network income using a random access algorithm is higher than that using the proposed one when the number of users is small. If the number of users increases, the network income from the random access algorithm is lower than that from ours. The reason for this is that, when the number of users falls into the first situation, the network congestion rate is low, so users in the proposed algorithm tend to choose the cheapest network to receive service. Users do not consider the price parameter in the random access algorithm. From the users’ perspective, this is an optimal situation, as they get the required quality of service and pay less money. When the number of users falls into the second situation, the network congestion rate is higher. Therefore, if users want to receive better service, they need to pay more.

## 5. Conclusions

Various kinds of wireless networks are readily available, but users are usually concerned with service quality and pricing. To meet QoS requirements and save costs, access point selection needs to optimized. In this paper, an integrated network access algorithm that takes pricing and unascertained membership into account is proposed. Real-time changes in network bandwidth, load, the number of users, and the price of service is dynamically adjusted in accordance with the degree of unascertained membership. In this algorithm, these parameters affect a user’s choice of network. If this algorithm is employed, users can enjoy better service and pay less, and the network can also increase its income. Simulation results show that the proposed scheme controls user access, decreases network congestion, and increases network income with high loads and a high number of users.

## Figures and Tables

**Table 1:** Simulation parameters.

Parameter	Power/W	M_threshold/dB	S_hysteresis/dB	Price/$	Available Bandwidth/(Mbit.s^−1^)
Cellular network	2.0	2.5	0.4	5	0.3
Wireless network	0.2	2.5	0.4	5	1, 2, 3
